# Effect of Tenofovir on Nucleotidases and Cytokines in HIV-1 Target Cells

**DOI:** 10.1371/journal.pone.0078814

**Published:** 2013-10-25

**Authors:** Nabanita Biswas, Marta Rodriguez-Garcia, Sarah G. Crist, Zheng Shen, Jack E. Bodwell, John V. Fahey, Charles R. Wira

**Affiliations:** Department of Physiology and Neurobiology, Geisel School of Medicine at Dartmouth, Lebanon, New Hampshire, United States of America; Salute San Raffaele University School of Medicine, Italy

## Abstract

Tenofovir (TFV) has been widely used for pre-exposure prophylaxis of HIV-1 infection with mixed results. While the use of TFV in uninfected individuals for prevention of HIV-1 acquisition is actively being investigated, the possible consequences of TFV exposure for the HIV-target cells and the mucosal microenvironment are unknown. In the current study, we evaluated the effects of TFV treatment on blood-derived CD4^+^ T cells, monocyte-derived macrophages and dendritic cells (DC). Purified HIV-target cells were treated with different concentrations of TFV (0.001-1.0 mg/ml) for 2 to 24hr. RNA was isolated and RT-PCR was performed to compare the levels of mRNA expression of nucleotidases and pro-inflammatory cytokine genes (MIP3α, IL-8 and TNFα) in the presence or absence of TFV. We found that TFV increases 5’-ecto-nucleotidase (NT5E) and inhibits mitochondrial nucleotidase (NT5M) gene expression and increases 5’ nucleotidase activity in macrophages. We also observed that TFV stimulates the expression and secretion of IL-8 by macrophages, DC, and activated CD4^+^ T cells and increases the expression and secretion of MIP3α by macrophages. In contrast, TFV had no effect on TNFα secretion from macrophages, DC and CD4^+^ T cells. Our results demonstrate that TFV alters innate immune responses in HIV-target cells with potential implications for increased inflammation at mucosal surfaces. As new preventive trials are designed, these findings should provide a foundation for understanding the effects of TFV on HIV-target cells in microbicide trials.

## Introduction

Three decades after the discovery of HIV, with no preventive vaccine available and limited success with microbicides, the HIV pandemic continues to spread with more than 30 million people living with HIV and almost 2.5 million new infections in 2011 [1]. Microbicides are compounds that can be applied topically and/or taken orally to prevent sexual transmission of HIV and other sexually transmitted infections (STI) [2]. One example of a microbicide is tenofovir (TFV), which is widely used for HIV-1 treatment and has been recently tested in clinical trials as a PrEP (Pre-Exposure Prophylaxis) [3]. TVF can be taken orally or administered topically into the vagina in a gel form. The CAPRISA 004 trial (Centre for the AIDS Program of Research in South Africa) tested the efficacy of the vaginal gel containing TFV in preventing HlV-1 acquisition, and found a 39% reduction in infection when the gel was applied before and after sexual intercourse [4]. In contrast, the VOICE (Vaginal and Oral Interventions to Control the Epidemic) trial, in which the TFV gel was applied to the vagina or taken orally on a daily basis, was discontinued due to lack of efficacy [5]. Although lack of adherence was a main factor involved in the failure of the VOICE trial, the possible contribution of changes in the biology of HIV-target cells induced by TFV needs to be explored.

TFV is a nucleotide analogue reverse transcriptase inhibitor [6,7]. After entering the cell, TFV requires two phosphorylation steps to be activated into TFV-diphosphate (TFV-DP), the form of TFV with anti-HIV activity [8,9]. Once incorporated into the nascent viral cDNA, TFV-DP causes chain termination and thus inhibits viral replication [7,10]. The benefits of TFV are that it suppresses viral replication, has a favorable safety profile and a relatively long half-life [11].

 5’-nucleotidases are a group of cytosolic enzymes that regulate the pool of cellular nucleotide and nucleoside levels by catalyzing the dephosphorylation of nucleoside monophosphate [12]. Seven 5’-nucleotidases have been isolated and characterized of which five are localized in the cytoplasm (NT5C1A, NT5C1B, NT5C2, NT5C3, NT5C3L), NT5E is bound to the extracellular part of the plasma membrane, and NT5M is present in the mitochondrial matrix [13]. Some of these enzymes are ubiquitous while others have restricted tissue distribution, however, the detailed expression profile of 5’-nucleotidases in human immune cells remains unknown. As suggested in clinical and in vitro studies, the activation of nucleoside analogs, such as TFV or emtricitabine, can be inhibited by an increase in nucleotidase activity [13]. However, the role that nucleoside analogs play in regulating 5’-nucleotidase expression and activity in HIV-target cells has not been explored. 

In addition to its anti-HIV-1 activity, TFV also possesses immunomodulatory properties by altering cytokine expression profiles in immune cells. It has been shown that TFV induces mRNA expression of IL-1β, TNFα, MIP1α/CCL3 and IL-10 cytokine in murine macrophages [14] and MIP1α/CCL3 and RANTES/CCL5 in human peripheral blood mononuclear cells (PBMC) [15]. Jesper and colleagues showed that after stimulation with Toll Like Receptor (TLR) ligands, TNFα, or live pathogens, TFV decreased IL-8 and CCL3 secretion by monocytes, and decreased CCL3 and IL-10 secretion by human PBMC [16]. As pro-inflammatory conditions in the Female Reproductive Tract (FRT) are associated with increased risk of HIV-1 transmission, it is important to evaluate the effects of TFV on cytokine and chemokine production by HIV-target cells.

In the present study, we investigated the possible immunomodulatory effects of TFV on HIV-target cells. We found that TFV was able to increase gene expression and secretion of cytokines and modify gene expression and bioactivity of 5’-nucleotidases in a time and dose-dependent manner. These studies provide insight into the actions of TFV on innate immune responses that should be informative when designing future HIV-prevention trials.

## Materials and Methods

### Study subjects

All investigations involving human subjects were carried out according to the principles expressed in the Declaration of Helsinki and carried out with the approval from the Committee for the Protection of Human Subjects (CPHS), Dartmouth Hitchcock Medical Center. Subjects were volunteer healthy donors recruited at Dartmouth Hitchcock Medical Center (DHMC) with written informed consent. 

### Generation of CD4^+^ T cells, Macrophages and Dendritic cells

Peripheral blood mononuclear cells (PBMC) were isolated using Ficoll density gradient centrifugation. CD14^+^ cells were positively selected using magnetic beads (Milteny Biotech, Auburn, CA, USA) to generate monocyte-derived macrophages. Cells were cultured for 4 days in an ultra-low attachment flask (Corning, Corning, NY, USA) with X-vivo 15 media (Lonza, Walkersville, MD, USA) supplemented with 10% human serum (Valley Biomedical, Winchester, VA, USA) [17]. Monocyte-derived dendritic cells were generated from monocytes as previously reported [18,19]. Briefly, after the isolation of CD14+ monocytes, final concentrations of 1,000 units/ml IL-4 (Peprotech, Rocky Hill, NJ, USA), and 1,000 units/ml of GM-CSF (Peprotech) were added at day 0 and supplemented every other day. Cells were cultured in X-vivo 15 media (Lonza, Walkersville, MD, USA) supplemented with 1% human serum (Valley Biomedical). After 5 days, cells were washed and plated in a 24 well plate (Corning). The purity of the cells was determined using flow cytometry with commercially labeled monoclonal antibodies (mAbs) against surface markers CD3, CD19 and CD56 (eBioscience, San Diego, CA) as mentioned previously [18,20]. 

CD4^+^ T cells were isolated from PBMC using magnetic negative selection (Milteny Biotech). Resting CD4^+^ T cells were plated immediately after isolation and were cultured in X-vivo 15 media (Lonza, Walkersville, MD, USA) supplemented with 10% human serum. To obtain activated CD4^+^ T cells, cells were supplemented with Phytohemagglutinin (PHA) (2.5 µg/ml; Sigma, St Louis, MO, USA) and IL-2 (50U/ml) (AIDS Research and Reference Reagent Program, Division of AIDS, NIAID, NIH: Human rIL-2 from Dr. Maurice Gately, Hoffmann- La Roche Inc) [21] for 24hr. 98% purity was obtained after selection of CD14+ monocytes and CD4^+^ T cells (data not shown). 

### Cell Viability assay

Viability of CD4^+^ T cells, Macrophages and Dendritic cells upon treatment with TFV was quantified using the CellTiter 96^®^ AQ_ueous_ One Solution Cell Proliferation assay (Promega, Madis, WI, USA) according to the manufacturer’s instructions. Briefly, reagent was added directly to cell cultures and was incubated for 1 hr at 37°. After 1hr absorbance of each well was read at 490 nm in a plate reader. Cell viability was also measured using Trypan blue (HyClone Laboratories, Inc; Logan, UT) method.

### Tenofovir (TFV) treatment

TFV in powder form was obtained from AIDS Research and Reference Reagent Program (NIH AIDS Reagent Program, Division of AIDS, NIAID, NIH: Tenofovir, catalog number 10199). A stock concentration of TFV (10mg/ml) was prepared by adding 1ml of PBS to 10mg of TFV powder. For most of the experiments presented, the final concentration of TFV used was 1mg/ml. For some experiments, a final concentration of TFV (0.5, 0.1, 0.01 and 0.001mg/ml) was also used. 

### RNA isolation and quantitative RT-PCR analysis

Real time PCR was done with a two-step protocol as described previously [22,23]. Using RNeasy reagent (Qiagen, Valencia, CA, USA) and QIAshredder column (Qiagen) total RNA was isolated according to the manufacturer’s recommendations. RNA was purified with RNeasy columns (Qiagen, Valencia, CA, USA) with on-column DNase digestion using the RNase-Free DNase set (Qiagen). 400ng of total RNA from each specimen was reverse-transcribed using the iScript cDNA synthesis kit (Bio-Rad, Hercules, CA, USA) in 20μl volume according to the manufacturer’s recommendations. 5’-Nucleotidases measured were Ecto-5’-nucleotidase (NT5E), Cytosolic 5’-nucleotidase 1A (NT5C1A), Cytosolic 5’-nucleotidase 1B (NT5C1B), Cytosolic 5’-nucleotidase II (NT5C2), Cytosolic 5’-nucleotidase III (NT5C3L), Cytosolic 5’(3’)-deoxyribonucleotidase (NT5C), and Mitochondrial 5’(3’)-deoxyribonucleotidase (NT5M). Relative mRNA expression of the 7 nucleotidase genes NT5E (Hs01573922_m1), NT5C1A (Hs00261369_m1), NT5C1B (Hs00403674_m1), NT5C2 (Hs00366992_m1), NT5C3L (Hs00369454_m1), NT5C (Hs01105359_g1), NT5M (Hs00220234_m1) and pro-inflammatory cytokine genes MIP3α (Hs00171125_m1), IL-8 (Hs00174103_m1) and TNFα (Hs011113624_g1) were measured by 5’-fluorogenic nuclease assay in real-time quantitative PCR using TaqMan chemistry on the ABI 7300 Prism real-time PCR instrument (Applied Biosystems, Foster City, CA, USA). GAPDH (Hs02758991_g1) primers were used to amplify GAPDH gene that was used as a housekeeping gene for macrophage and dendritic cells. For CD4^+^ T cells RPL13A (Hs003043884_g1) was used as a housekeeping gene [24]. PCR was done using the following cycle parameters: 95°C, 12 min for 1 cycle (95°C, 20 s; 60°C, 1 min), for 40 cycles. Analyses were performed using sequence detection software supplied with the ABI 7300. This software calculates the threshold cycle (C_t_) for each reaction, which was used to quantify the amount of starting template in the reaction. The average of C_t_ values for each set of duplicate reactions was calculated and a difference in C_t_ values (ΔC_t_) was determined for each gene by taking the mean C_t_ of each gene of interest and subtracting the mean C_t_ for the housekeeping gene GAPDH/RPL13A for each cDNA sample. Assuming that each reaction functions at 100% PCR efficiency, a difference of one C_t_ represents a 2-fold difference. Relative expression of genes were calculated using the formula 2^−ΔCt^. 

### Measurement of 5’-Nucleotidase biological activity assay

5’-nucleotidase biological activity was measured using 5’-nucleotidase kit (Diazyme Laboratories, Poway, CA, USA) that was modified to determine the effect of TFV on 5’-nucleotidase (NT) biological activity as described previously [25]. The assay was adapted from a serum to a cellular based assay by modifying the manufacturer’s protocol. Briefly, 0.4×10^6^ macrophages, 1×10^6^ activated or resting CD4^+^ T cells were treated with PBS (control) and TFV (1mg/ml) for 24 and 48hr. Cells were washed with Hepes Buffered Saline (0.15M NaCl, 20mM HEPES, pH 7.4) and were dislodged with cell stripper (Cellgro, Manassas VA, USA). Cells were permeabilized using Hepes Buffered Saline containing 7.5mM CHAPS (Research Organics, Cleveland, OH, USA) by incubating the cells on ice for 20min (CD4^+^ T cells) to 1hr (macrophages). Permeabilized cells (20μl) were added to the 96 well plate along with appropriate kit reagents (Reagents 1 and 2). Absorbance was monitored at 550nm (25°C) on a SpectraMax, M5 (Molecular Devices Corporation, Sunnyvale, CA, USA). Changes in absorbance were determined at 1 min intervals for ~15min and results over a seven-minute interval were averaged once the baseline was stabilized. A molar extinction coefficient of 18440 M^-1^ cm^-1^ was used to calculate the enzyme activity (1 unit is equal to the production of 1mmole of quinone dye/min) and standards were run with each assay to verify assay linearity. Data were expressed as activity per million cells.

### ELISA assays

Secretion of cytokines (MIP3α, IL-8 and TNFα) in cell culture was measured by enzyme-linked immunosorbent assay (ELISA). TFV (1mg/ml) was added to macrophages, DCs and activated and resting CD4^+^ T cells for 12, 24 and 48hr. Supernatants were recovered and centrifuged (10,000g) for 10min to remove cellular debris. MIP3α, IL-8 and TNFα (all from R&D Systems, Minneapolis, MN, USA) were measured by ELISAs according to the manufacturer’s instructions. Protein concentration was quantified based on a standard curve after OD measurements at 450 and 570nm on an ELISA reader (Dynex, Chantilly, VA, USA).

### Statistical analysis

GraphPad Prism 5.0 software was used to analyze the data. To compare between two groups, non-parametric test, U-Mann Whitney or Wilcoxon paired tests were performed. Non-parametric Kruskal-Walis test followed by Dunns post-test was done for comparing three or more groups. A two sided P value < 0.05 was considered statistically significant in all cases. 

## Results

### Dose-dependent effects of Tenofovir on the expression of nucleotidase genes in macrophages

**Figure 1:**
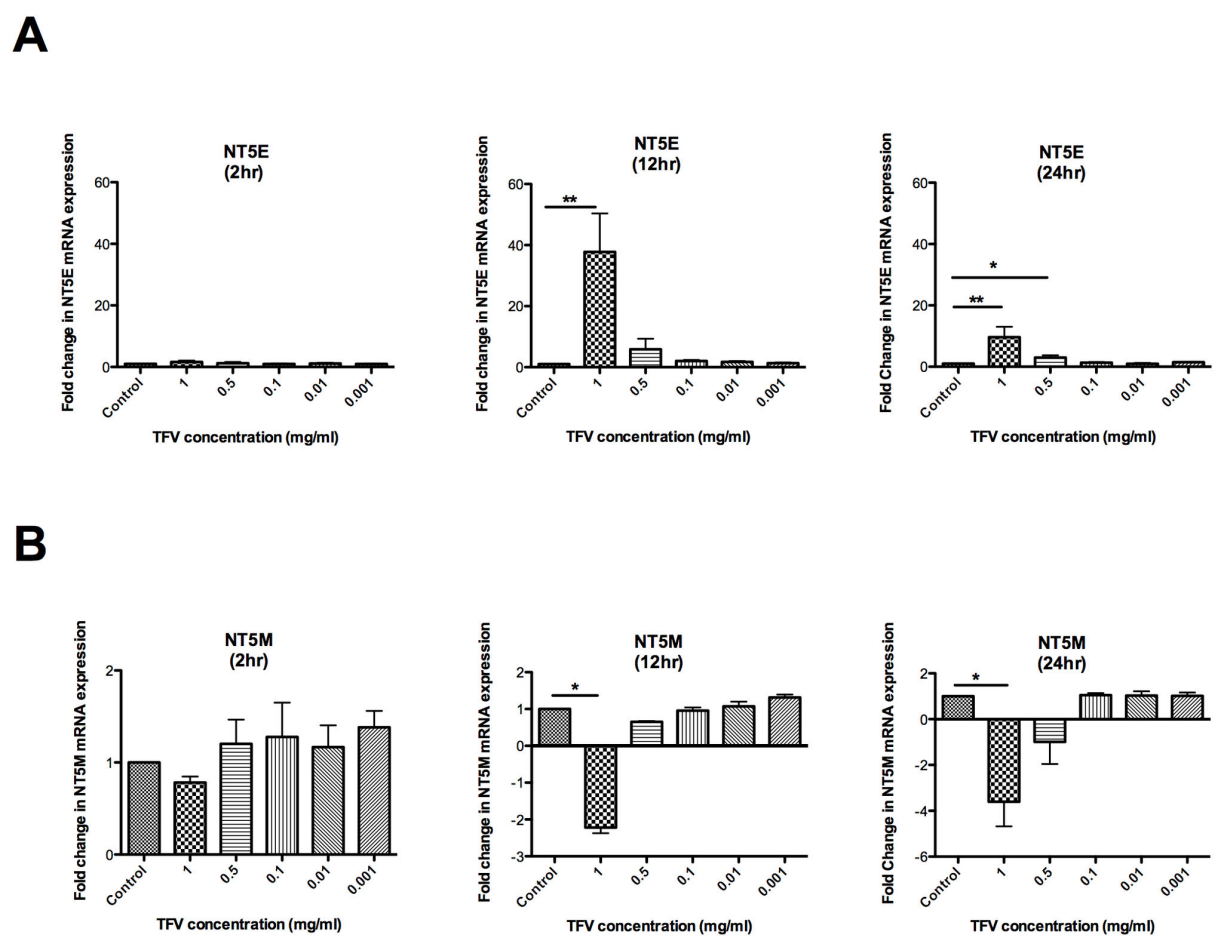
Dose-dependent effects of Tenofovir on the expression of nucleotidase genes in macrophages. Monocyte-derived macrophages were treated with different concentrations of TFV (1, 0.5, 0.1, 0.01, 0.01 and 0.001 mg/ml) for 2, 12 and 24hr. RNA was isolated and RT-PCR was performed to compare the levels of mRNA of NT5E and NT5M with different concentration of tenofovir at different time points. mRNA was expressed as a fold change over untreated samples (assigned a value of 1). Bars represent mean ± SEM from 4 separate experiments with different donors. *P<0.05 **P<0.01. (**A**) Macrophage expression of ecto-5’-nucleotidase (NT5E) gene in presence of different concentrations of tenofovir at 2, 12 and 24hr. (**B**) Macrophage expression of mitochondrial 5’(3’)-deoxyribonucleotidase gene (NT5M) in presence of different concentrations of tenofovir at 2, 12 and 24hr.

To determine the effect of TFV on the mRNA expression of nucleotidase genes in Monocyte Derived Macrophages (N=4 patients), dose-response experiments were performed with purified macrophages (>98%) using 1, 0.5, 0.1, 0.01 or 0.001mg/ml of TFV for 2, 12 and 24hr. RNA was isolated from the cells and real-time PCR was done to analyze the effect of TFV on the mRNA expression of 5’-nucleotidase genes. The expression of six out of seven nucleotidase genes analyzed was detectable in macrophages. NT5C1B was undetected. The expression of NT5E and NT5M changed significantly following treatment with TFV. As shown in Figure **1A**, although no significant change was observed in the NT5E gene expression at 2hr (Figure **1A**;**left panel**), a significant increase in the gene expression of NT5E was observed at 12hr (Figure **1A** **;middle panel**) (mean fold increase 38) and 24hr (**right panel**) (10-fold) with a TFV dose of 1mg/ml. Additionally, a significant 3-fold increase in NT5E gene was observed at 24hr (Figure **1A** **;right panel**) with 0.5mg/ml TFV. 

In contrast to the increase in NT5E expression, a significant dose-dependent decrease (Figure **1B**) in the expression of the NT5M was observed at 12hr (Figure **1B** **;middle panel**) (-2.0-fold) and 24hr (Figure **1B** **;right panel**) (-3.0-fold) with a TFV dose of 1mg/ml. These results suggest that TFV can specifically modulate the expression of nucleotidase genes and that expression of these genes depends on incubation time and the concentration of TFV. Since 1mg/ml gave us the most significant results, we selected this concentration for subsequent experiments.

### Time-dependent effects of Tenofovir on the expression of nucleotidase genes in macrophages, dendritic cells and CD4^+^ T cells

**Figure 2:**
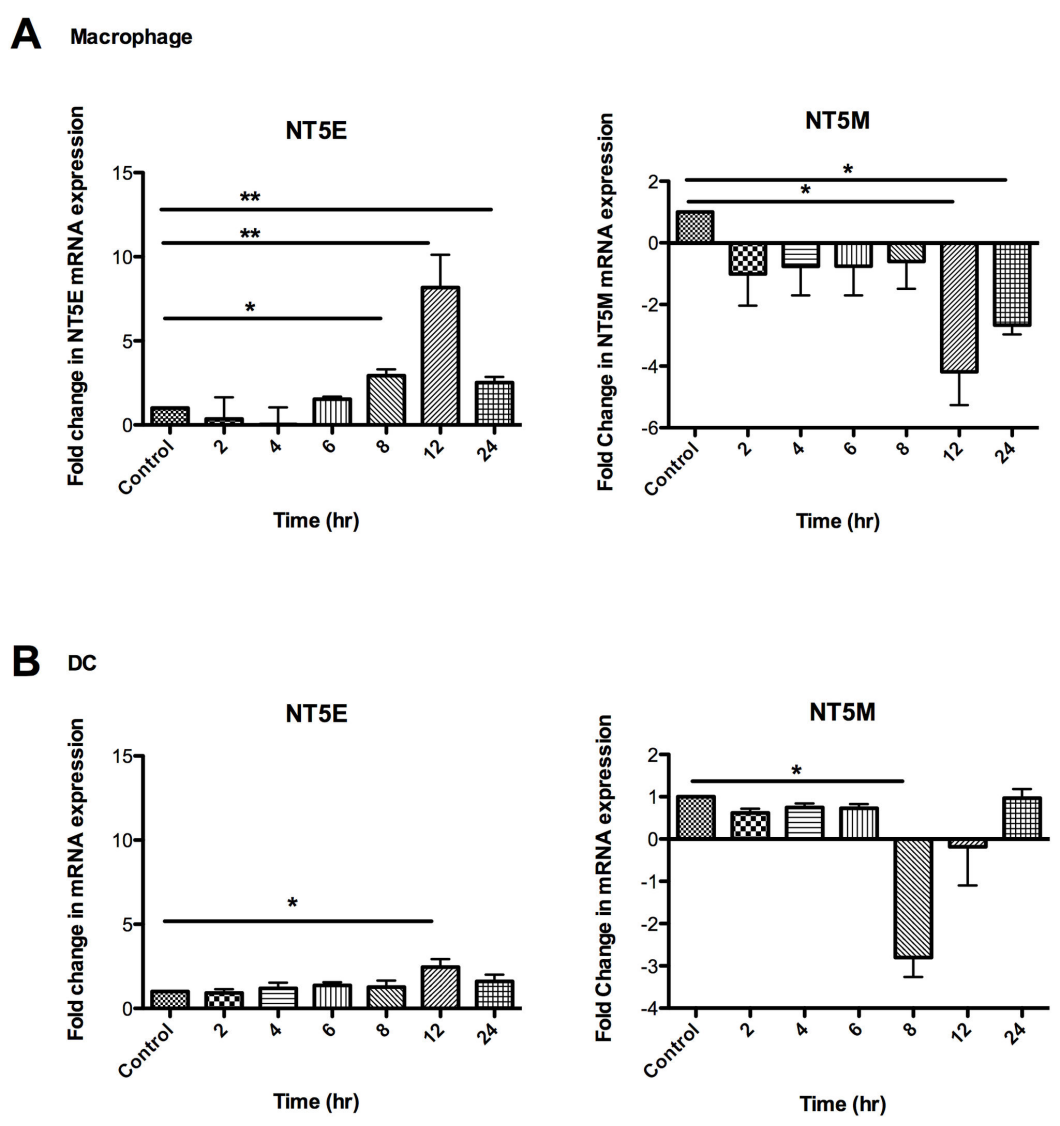
Time-dependent Effects of Tenofovir on the expression of nucleotidase genes in macrophages, dendritic cells and activated and resting CD4^+^ T cells. Monocyte-derived macrophages were treated with 1mg/ml of TFV for 2 to 24hr. After 3 days cells were treated with 1mg/ml of TFV for 2 to 24hr. Bars represent mean ± SEM from 4 separate experiments with different donors. *P<0.05 **P<0.01. (A) Macrophage expression of ecto-5’-nucleotidase (NT5E) and mitochondrial 5’(3’)-deoxyribonucleotidase nucleotidase gene (NT5M) gene at different time points . (B) DC expression of ecto-5’-nucleotidase (NT5E) and mitochondrial 5’(3’)-deoxyribonucleotidase nucleotidase gene (NT5M) gene at different time points .

Recognizing that TFV increases the gene expression of NT5E and inhibits the gene expression of NT5M in macrophages, we wanted to more fully examine the extent to which TFV regulates nucleotidase gene expression in blood-derived macrophages, DCs, and activated and resting CD4^+^ T cells in detailed time courses. Cells were treated with 1mg/ml of TFV for 2 to 24hr. Similar to our findings with macrophages, DCs and resting and activated CD4^+^ T cells expressed six out of seven nucleotidase genes. NT5C1B was undetected in all cells. Significant changes were observed in the expression of nucleotidase genes NT5E and NT5M in macrophages (Figure **2A**) and in DCs (Figure **2B**). Figure **2A** shows the expression of NT5E and NT5M genes in macrophages with a detailed time course. A significant 3.0-fold increase in the expression of NT5E was observed at 8hr, with a maximum expression at 12hr (8.0-fold) and a 3.0-fold increase at 24hr **(** Figure **2A**;**left panel**). In contrast, a significant decrease in NT5M gene expression was observed at 12hr (-4.0-fold) and at 24hr (-3.0-fold) (Figure **2A**;**right panel**). For DCs, a significant increase (2.0-fold) in the NT5E gene was observed at 12hr after adding TFV **(** Figure **2B**;**left panel**). For NT5M gene expression, a significant decrease (-3.0-fold) was observed at 8hr (Figure **2B**;**right panel**). In the case of resting and activated CD4^+^ T cells, no significant changes were observed in the expression of nucleotidase genes in the presence of TFV (data not shown). Taken together these data suggest that modulation in the expression of NT5E and NT5M genes by TFV is time-and cell type-dependent.

A lower dose of TFV (0.1mg/ml) was used for 2-24hr on activated and resting T cells but we did not observe any significant changes in gene expression compared to the control (data not shown).

### Effect of Tenofovir on 5’-Nucleotidase biological activity in resting and activated CD4^+^ T cells and in macrophages

**Figure 3:**
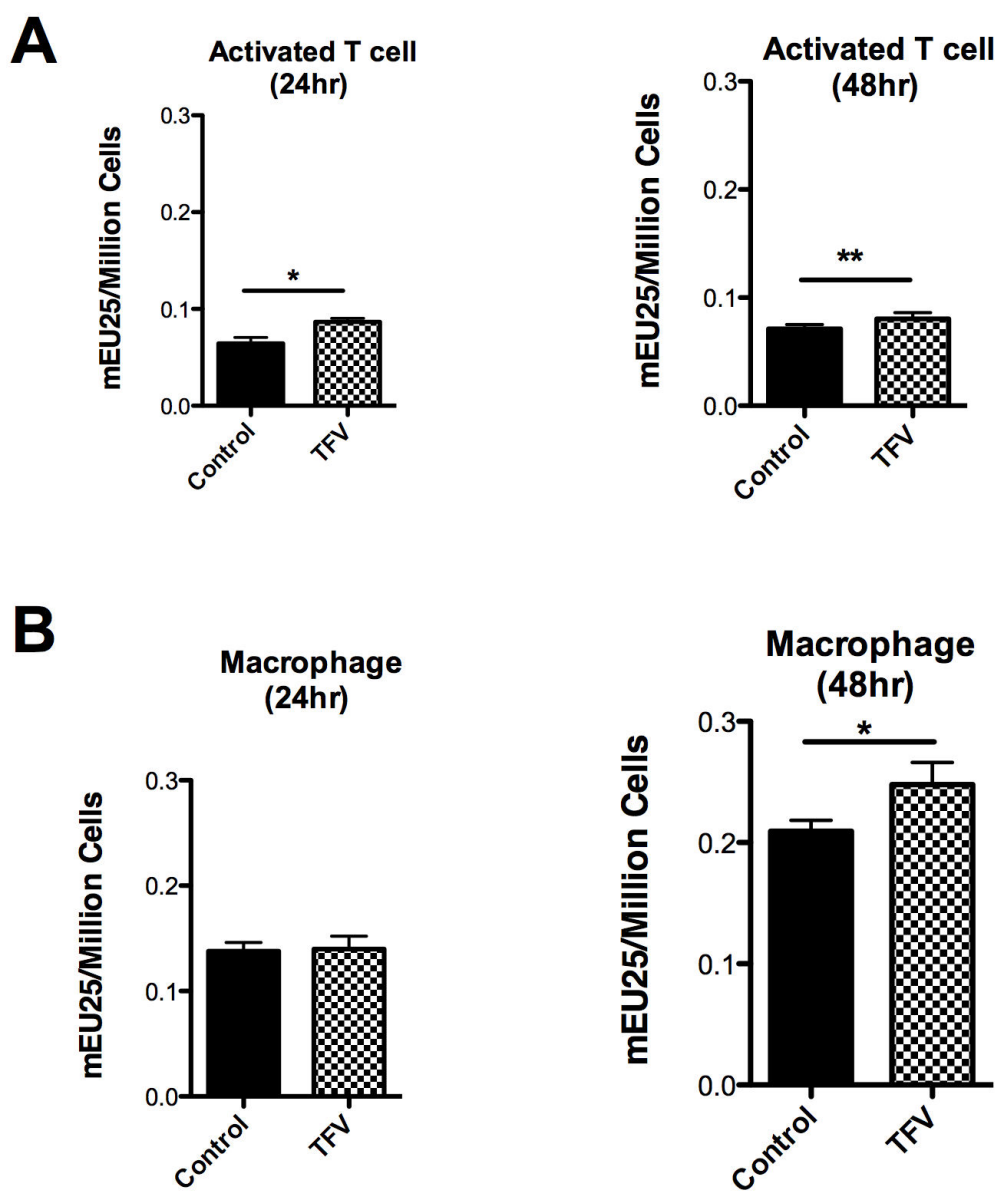
Effect of Tenofovir on nucleotidase biological activity in activated CD4^+^ T cells and macrophages. 5’-Nucleotidase biological activity was measured (see methods section) in activated CD4^+^ T cells (**A**) and in macrophages (**B**) after 24 and 48hr with 1mg/ml of TFV treatment. Values are expressed as milli-Enzyme Units (mEU) per one million cells. Bars represent mean ± SEM from 5 separate experiments with different donors. *P<0.05 **P<0.01.

Since changes in gene expression do not necessarily correlate with changes in enzymatic activity, studies were undertaken to determine if TFV modulates 5'-nucleotidase biological activity in resting CD4^+^ T cells, activated CD4^+^ T cells and in macrophages using a modified Diazyme 5’-Nucleotidase assay [25]. Activated CD4^+^ T cells (Figure **3A**) and macrophages (Figure **3B**), were treated with TFV (1mg/ml) and 5’-Nucleotidase activity was measured after 24 and 48hr. In the case of resting CD4^+^ T cells, no significant changes were observed in the 5’-nucleotidase activity in the presence of TFV compared to the control (data not shown). In contrast, a significant increase in the activity of 5’-nucleotidase in the presence of TFV was observed in activated CD4^+^ T cells at 24hr (p=0.039) **(** Figure **3A**;**left panel**) and at 48hr (p=0.0078) (Figure **3A**;**right panel**). 

In the case of macrophages, no significant changes were observed in the activity of 5’- nucleotidase in the presence of TFV at 24hr **(** Figure **3B**;**left panel**), but there was a significant increase at 48hr (p=0.0376) (Figure **3B** **;right panel**). 

### Dose-dependent effects of Tenofovir on the expression of MIP3α, IL-8 and TNFα genes in macrophages

**Figure 4:**
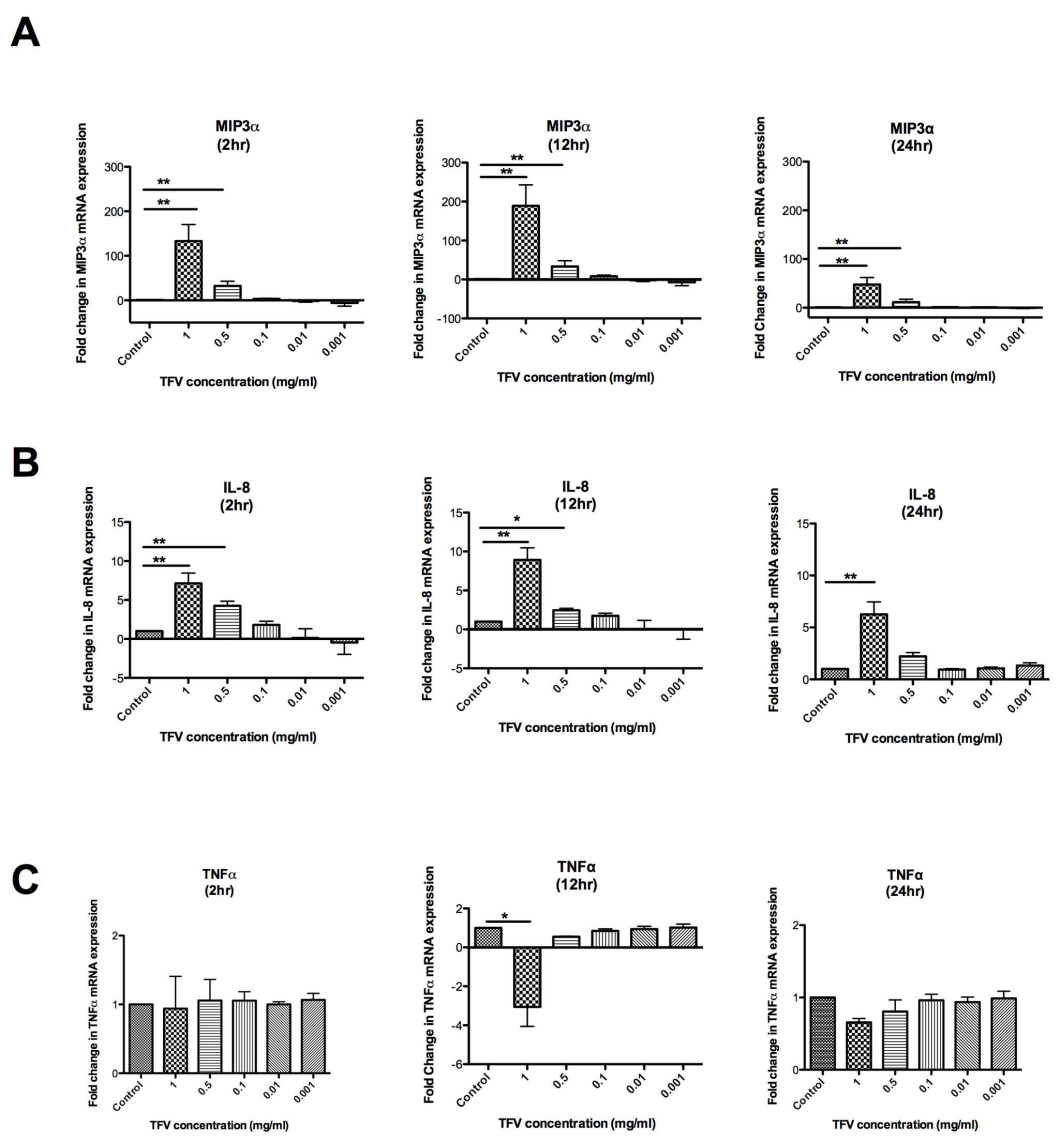
Dose-dependent effects of Tenofovir on the expression of MIP3α, IL-8 and TNFα genes in macrophages. Monocyte-derived macrophages (macrophages) were treated with different concentrations of TFV (1, 0.5, 0.1, 0.01, 0.01 and 0.001 mg/ml) for 2, 12 and 24hr. Bars represent mean ± SEM from 4 separate experiments with different donors. *P<0.05 **P<0.01. (**A**) Macrophage expression of MIP3α gene in presence of different concentrations of tenofovir at 2, 12 and 24hr. (**B**) Macrophage expression of IL-8 gene in presence of different concentrations of tenofovir at 2, 12 and 24hr. (**C**) Expression of TNFα gene in presence of different concentrations of tenofovir at 2, 12 and 24hr.

Recognizing that TFV can alter cytokine expression profiles in human PBMC [16], we next focused on whether TFV has any effect in the expression of cytokine and chemokines gene expression in HIV-1 target cells. To evaluate the effect of TFV on the mRNA expression of MIP3α, IL-8 and TNFα, macrophages were treated with 1, 0.5, 0.1, 0.01 or 0.001mg/ml TFV for 2, 12 and 24hr. RNA was isolated from the cells and real-time PCR was performed. As shown in Figure **4A**;**left panel**, a significant 133- and 32-fold increase in the expression of MIP3α mRNA was observed with 1mg/ml and with 0.5mg/ml TFV respectively at 2hr. A 189-fold increase of MIP3α mRNA was observed with 1mg/ml of TFV and 33-fold increase was observed with 0.5mg/ml of TFV at 12hr (Figure **4A** **;middle panel**). At 24hr, the increase in MIP3α mRNA was still maintained (47-fold) with 1mg/ml TFV and with 0.5mg/ml TFV (11-fold) (Figure **4A** **;right panel**). 

Similarly, a significant dose-dependent increase in the expression of IL-8 was also observed at 2hr (7-fold) (Figure **4B**;**left panel**), 12hr (9-fold) (Figure **4B** **;middle panel**) and 24hr (6-fold) (Figure **4B** **;right panel**) with a dose 1mg/ml of TFV. This increase in IL-8 mRNA expression was also observed with 0.5mg/ml TFV at 2hr (4-fold) and 12hr (3-fold) (Figure **4B** **;right panel**)**.**

In contrast to increases in MIP3α and IL-8 gene expression in the presence of TFV, there was a significant decrease in the expression of TNFα at 12hr (-3-fold) with 1mg/ml TFV (Figure **4C** **;middle panel**). No significant changes were observed at 2hr and 24hr. These results indicate that TFV can modulate the expression of cytokine genes in HIV-target cells and that expression of these genes depends on time and the concentration of TFV. 

### Time-dependent Effects of Tenofovir on the expression of MIP3α, IL-8 and TNFα genes in macrophages, dendritic cells, resting and activated CD4^+^ T cells

Based on our results that TFV (1mg/ml) induced the highest up-regulation in gene expression of MIP3α and IL-8 in macrophages, we next examined the effect of TFV on the different cell subsets in a detailed time course studies. Macrophages and DCs were treated with 1mg/ml TFV from 2 to 24hr and resting and activated CD4^+^ T cells were treated with TFV for 2, 12 and 24hr. RNA was isolated from the cells and real- time PCR was performed to analyze the effect of TFV on the mRNA expression of MIP3α, IL-8 and TNFα genes. 

**Figure 5:**
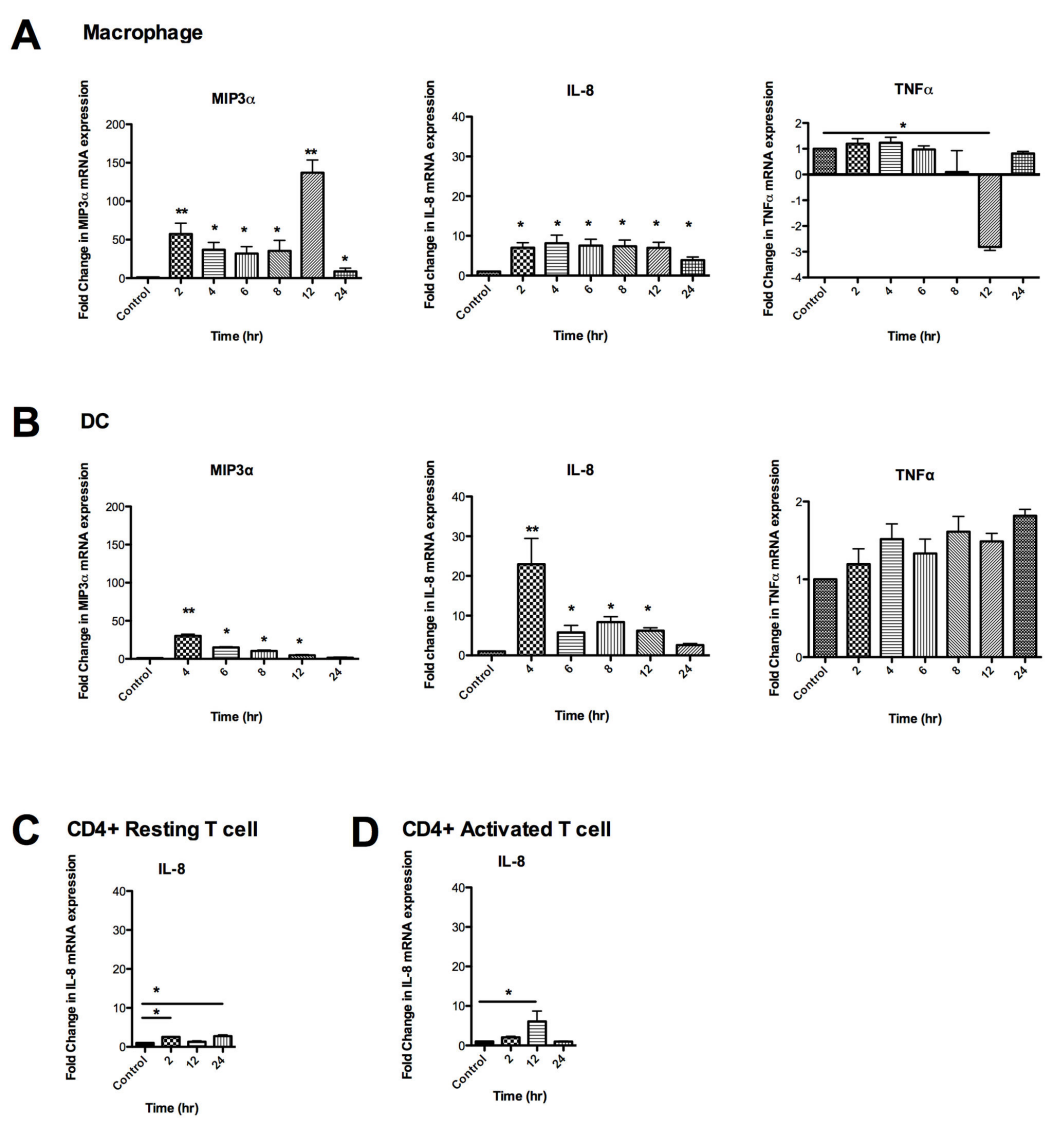
Time dependent effects of Tenofovir on the expression of MIP3α, IL-8 and TNFα genes in macrophages, dendritic cells and resting and activated CD4^+^ T cells. Macrophages and DCs were treated with 1mg/ml of TFV for 2 to 24hr. CD4^+^ resting and activated T cells were treated with 1mg/ml of TFV for 2, 12 and 24hr. Bars represent mean ± SEM from 3-4 independent experiments with different donors. *P<0.05 **P<0.01. (**A**) Macrophage expression of MIP3α, IL-8 and TNFα genes at different time points (**B**) DC expression of MIP3α, IL-8 and TNFα genes at different time points. (**C**) Expression of IL-8 gene at 2, 12 and 24hr in resting CD4^+^ T cells. (**D**) Activated CD4^+^ T cells expression of IL-8 gene at 2, 12 and 24hr.

As seen in Figure **5**, macrophages and DCs behave quite differently in their response to TFV treatment. While both macrophage and DCs significantly increased MIP3α mRNA message levels at the earliest time points (Figure **5A & 5B**;**left panels**), the highest peak in macrophages occurred at 12hr (137-fold). The earliest time point (4hr) assayed for DCs was the highest value (30-fold). The gradual decline at later time points in DCs suggests that the maximum for MIP3α mRNA may have occurred before 4hr. In contrast, there was no significant change in TNFα expression in DCs (Figure **5B** **;right panel**). We found that TFV inhibited by -3.0-fold TNFα expression in macrophages at 12hr (Figure **5A** **right panel**). TFV altered the expression of IL-8 message in DCs similarly to MIP3α in that the highest expression occurred at the earliest time assayed at 4hr (23-fold) (Figure **5B** **;middle panel**). In macrophages IL-8 message levels were elevated early (2hr, 7-fold) and remained elevated for 12hr and only partially returned towards baseline at 24hr (4-fold) (Figure **5A** **;middle panel**). 

The response of CD4^+^ T cells to TFV was quite different between resting and activated CD4^+^ T cells. IL-8 message was significantly enhanced at 12hr (6-fold, Figure **5D**) in activated CD4^+^ T cells. In contrast, the response was muted in resting CD4^+^ T cells and showed a biphasic stimulatory response at 2 (2.5 fold) and 24hr (2.7 fold) (Figure **5C**). 

### Effect of TFV on the secretion of MIP3α, IL-8 and TNFα by macrophages, DCs and resting and activated CD4^+^ T cells

**Figure 6:**
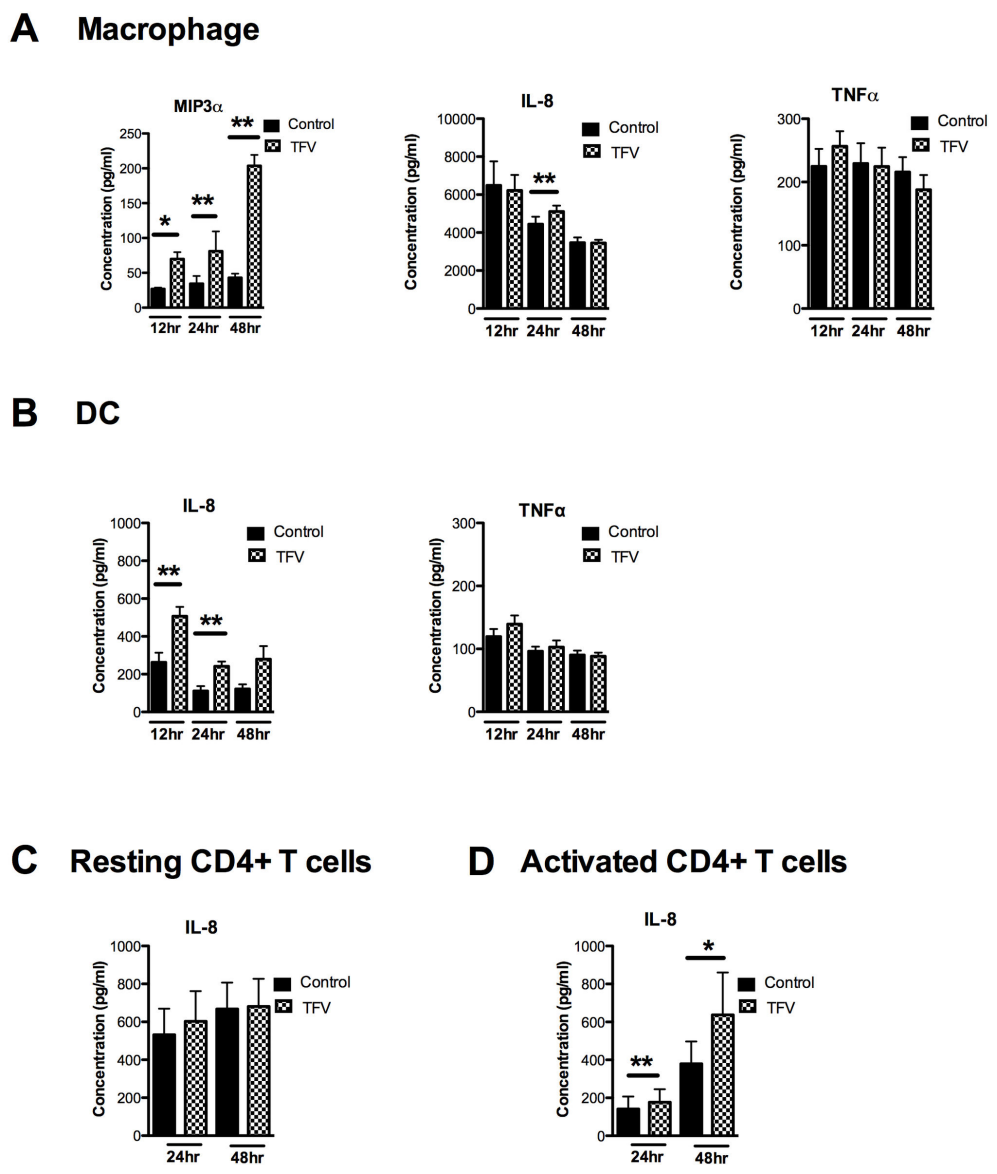
Effect of TFV on the secretion of MIP3α, IL-8 and TNFα by macrophages, DCs and resting and activated CD4^+^ T cells. Macrophages, DCs were treated with 1mg/ml of TFV for 12, 24 and 48hr and resting and activated CD4^+^ T cells were treated with TFV for 24 and 48hr. Secretion of MIP3α, IL-8 and TNFα was analyzed from culture supernatants by an enzyme-linked immunosorbent assay. Bars represent mean ± SEM from 3-4 independent experiments with different donors. *P<0.05 **P<0.01. **A**) secretion of MIP3α, IL-8 and TNFα from macrophages (**B**) secretion of IL-8 and TNFα from DCs. (**C**) secretion of IL-8 from resting CD4^+^ T cells. (**D**) secretion of IL-8 from activated CD4^+^ T cells.

To determine the effects of TFV on cytokine/chemokine proteins, we measured secretion of MIP3α, IL-8 and TNFα in the cell culture from macrophages, DCs and resting and activated CD4^+^ T cells. As seen in Figure **6**, incubation of macrophages with TFV (1mg/ml) significantly increased the secretion of MIP3α at 12 (p=0.0048), 24 (p=0.040) and 48hr (p=0.019) relative to control cells (Figure **6A**;**left panel**). Secretion of IL-8 increased significantly at 24hr (p=0.0068) (Figure **6A** **;middle panel**) after which no additional differences were seen. At no time were changes observed in the secretion of TNFα (Figure **6A** **;right panel**)**.**

When DCs were incubated with TFV, we found a significant increase in IL-8 secretion at 12 (p=0.01), 24 (p=0.01) (Figure **6B**;**left panel**). No differences were observed in TNFα secretion (Figure **6B** **;right panel**); MIP3α secretion was below the limit of detection of the assay (data not shown). 

As seen in Figure **6C**, TFV had no effect on IL-8 secretion by resting CD4^+^ T cells. Both TNFα and MIP3α secretion were below the limit of detection in the assay (data not shown). In contrast, activated CD4^+^ T cell secretion of IL-8 increased significantly at 24 (p=0.0078) and 48hr (p=0.03) in the presence of TFV (Figure **6D**). Similar to resting CD4^+^ T cells, activated CD4^+^ T cell secretion of TNFα and MIP3α was below the limit of detection (data not shown). 

## Discussion

The present study investigated the effects of TFV with respect to its ability to modulate 5’-nucleotidase gene expression, nucleotidase biological activity and chemokine production by CD4^+^ T cells, macrophages and DCs, the main target cells for HIV-1 infection. We found that TFV altered expression of two of the seven 5’-nucleotidase genes, NT5E (CD73) and NT5M, but had no effect on any of the intracellular 5’-nucleotidases. TFV specifically increased NT5E and decreased NT5M gene expression in macrophages and DCs, with no changes in message observed in CD4^+^ T cells. As a part of these studies, we found that TFV increased IL-8 secretion in all cell types analyzed, and increased MIP3α production in macrophages and DCs. The stimulatory effect of TFV on nucleotidase gene expression and chemokine secretion suggests a link between TFV treatment and the recruitment of immune cells to the FRT that may compromise microbicide effectiveness against HIV infection in the FRT.

In the present study, we demonstrate that 5’-nucleotidase gene expression and biological activity is up-regulated by TFV treatment of macrophages. In contrast, we found a lack of correlation between 5’-nucleotidase mRNA levels and biological activity in CD4^+^ T cells, which could be due to auto-regulatory mechanisms at the transcriptional level [26]. The increase in the NT5E gene expression in the presence of TFV was not associated with the cell viability. Using trypan blue method cell viability was analyzed and no difference was observed in the cell viability when it was compared with control. Our findings are consistent with previous reports by Christensen et al describing a disconnection between CD73 gene expression and NT5E bioactivity in blood mononuclear cells [27]. Another possible explanation for the lack of correlation seen in our study is that our modified nucleotidase biological activity assay does not detect all of the different nucleotidase forms equally. For example, others have shown that this assay detects the activity of nucleotidases that prefer purine based substrates, such as NT5E, NT5C1A, and NT5C2, which are the 5’-NT expected to interact with TFV as a purine analog. Nucleotidases with a preference for pyrimidine bases, such as NT5C, NT5C3 and NT5M, would contribute only marginally to the activity detected with this assay [25]. Our studies expand the findings of Shen et al who showed that estradiol increases nucleotidase biological activity by demonstrating that TFV in selected cells also upregulates nucleotidase gene expression and biological activity [25].

5',3'-nucleotidase, mitochondrial (NT5M) is localized in the mitochondrial membrane [28]. Our studies have demonstrated that TFV inhibits the expression of NT5M in macrophages and in DCs. Others have shown that several antiviral nucleosides, including AZT, deplete mitochondrial DNA with side effects consistent with mitochondrial toxicity [29]. Dephosphorylation of AZT and other analogs by NT5M would be expected to reduce the toxic side effects of the drug in the mitochondria. Our studies extend these findings by demonstrating that TFV inhibits the expression of NT5M in macrophages and in DCs. Further, they suggest that TFV may accumulate in these cells and therefore be more likely to compromise mitochondrial function. 

To further understand the mechanism of TFV action, we examined the effect of TFV on chemokine production and found that TFV increases MIP3α in macrophages and DCs, and increases IL-8 production in all cell types analyzed. These effects occur at a time when TNFα expression is not affected. Since changes in message were detected as early as 2h after TFV exposure, MIP3α and IL-8 are likely directly up-regulated by TFV. The implications for HIV-acquisition of MIP3α and IL-8 stimulation by TFV are complex and important at several levels. For example, Ghosh et al previously demonstrated that MIP3α has potent anti-HIV-1 activity *in vitro* [30]. In other studies, MIP3α was shown to up-regulate the host restriction factor APOBEC3G [31], which inhibits HIV-1 infection in the absence of the viral protein Vif [32]. In contrast to its beneficial effects, MIP3α is known to be chemotactic for HIV-target cells including dendritic cells and CD4^+^ T cells [33,34]. Studies in macaques demonstrated that blockade of MIP3α prevented systemic dissemination of SIV-infection following vaginal challenge [35]. In addition to MIP3α, IL-8 is also a chemoattractant molecule for CD4^+^ T cells as well as neutrophils [36]. Moreover, IL-8 can increase HIV replication in macrophages and CD4^+^ T cells, and has been shown to increase viral replication in ectocervical explants and peripheral blood lymphocytes [36-38]. Our findings suggest, that since TFV rapidly induces MIP3α and IL-8 secretion by macrophages, DCs and activated CD4^+^ T cells, it may increase susceptibility to HIV-1 infection by amplifying the presence of HIV-1 target cells at the genital mucosal, in which case timing of TFV application and sexual contact becomes a critical issue.

In contrast to the rapid increases in chemokine genes observed in our study, up-regulation for NT5E mRNA was measurable initially at 12h following TFV exposure, suggestive of an indirect mechanism of up-regulation. Others have reported that cytokines can regulate the expression of 5’-nucleotidase genes. For example, TNFα increases the expression of NT5E in PBMC [39], and IL-4 is known to decrease NT5E activity in lymphocytes and monocytes [40,41]. It was also reported that IFN-γ reduces NT5E activity in monocytes [40,41]. Further studies are needed to determine if the rapid effects of TFV on MIP3α, IL-8 production, and other chemokines and cytokines not analyzed in our study, are responsible for the expression of NT5E in macrophages and in DCs. 

HIV-preventive trials testing TFV taken orally or in gel form have yielded mixed results. Partial success was achieved in the CAPRISA 004 study, using 1% TFV gel before and after sex, where 39% protection against HIV-1 was observed [4]; and in the iPrex trial, daily doses of FTC-TDF (emtricitabine- tenofovir disoproxil fumarate) resulted in 44% protection among men or transgender women who have sex with men [42]. In the VOICE trial, however, the oral and vaginal arms of the study were stopped due to lack of effect, explained by a lack of adherence [5]. The results presented in our studies are biologically relevant given that several trials are underway to test the efficacy of TFV in preventing HIV-infection. This includes the FACTS 001 trial [43], designed to reproduce the results from the CAPRISA 004 study, using TFV gel before and after sex. Future studies are considering the use of vaginal rings containing TFV and contraceptives to by-pass the adherence problems [44]. Although much effort is being invested in testing the efficacy of TFV in preventing HIV infection, little is known about the consequences of TFV exposure in the mucosal microenvironment and the HIV-target cells. 

In the present study, we used different doses of TFV, with 1mg/ml being the highest concentration of TFV. We found no evidence of cell death but found significant alterations in the biology and immune responses of HIV-target cells. These findings indicate that TFV is not a null substance for immune cells. Just how the doses of TFV used in our study correlate to the doses used in TFV trials remains to be determined. In the CAPRISA 004 trial, one dose of gel contained 40mg of TFV and was used before and after sex, resulting in vaginal deposition of 80mg in 24h [4]. In the iPrex trial a combination of two drugs: 300mg of Viread (tenofovir DF) and 200mg of Emtricitabine (FTC) were used [42]. While several studies have measured tissue levels [45], correlations between the amount of TFV administered and the concentration of antivirally active TFV-DP in immune cells and in the female reproductive tract is still being investigated. Our studies suggest that concentrations below 0.1mg/ml do not elicit proinflammatory cytokine/chemokine responses in HIV-target cells. In other studies (Shen et al, unpublished data) we found that concentrations as low as 0.001mg/ml are effective in preventing HIV infection. Further studies are needed to identify the amount of TFV that reaches immune cells to optimize protection without triggering responses that compromise immune protection.

In conclusion, our study demonstrates that TFV regulates gene expression and biological activity of 5’-nucleotidases and production of MIP3α and IL-8 in macrophages, DCs and CD4^+^ T cells, the main targets for HIV-infection. These findings should contribute to the foundation of understanding on how TFV may modulate immune protection in HIV-target cells in the FRT. As new preventive trials are designed, more information is needed to fully understand the effects of TFV in HIV-target cells in the female reproductive tract. 
